# Multimodal stone therapy for two forgotten and encrusted ureteral stents: a case report

**DOI:** 10.1186/1757-1626-2-106

**Published:** 2009-01-30

**Authors:** Thorsten H Ecke, Steffen Hallmann, Jürgen Ruttloff

**Affiliations:** 1 Department of Urology, HELIOS Hospital, Pieskower Str 33, Bad Saarow 15526, Germany

## Abstract

**Introduction:**

Ureteral stent placement is a common procedure in daily urologic practice. To manage the problems of forgotten stents for many years needs multimodal stone therapy.

**Case presentation:**

We present a case of a 26-years-old Caucasian, white woman with two forgotten encrusted ureteral stents for 48 months. Multimodal stone therapy including extracorporeal shock wave (SWL), percutaneous nephrolithotomy (PCNL), ureterorenoscopy (URS), cystolithotripsy with Lithoclast, and open surgery was necessary to remove all the stones. Using the described combination of techniques, our patient was rendered stone and stent free. Urologists should bear in mind the presence of severe encrustations when they have to deal with a forgotten stent.

**Conclusion:**

This case shows that combined urologic techniques can achieve successful and safe management of forgotten stents, but treatment should be tailored to the volume of encrustation and associated stone.

## Introduction

Ureteral stent placement is a common procedure in daily urologic practice. The indications include relief of ureteral obstruction of diverse etiologies, ensuring adequate postoperative drainage, and prevention of ureteral injuries during surgical procedures. During the last decade, significant technological innovations and improvements have been made in stent design and material in order to overcome problems related to stent manipulation and patient tolerance [1,2]. Serious complications including migration, fragmentation, encrustation, and stone formation, still occur, especially when stents have been forgotten for a long time [3,4].

Previous studies of retained and encrusted stents have recognized the associated risk of serious morbidity and have introduced endoscopic management [3,5-9].

Forgotten ureteral stents represent a difficult problem in urology. Combinations of extracorporeal shockwave lithotripsy (SWL), ureterorenoscopy (URS), electrohydraulic lithotripsy, laser lithotripsy, and percutaneous nephrolithotomy (PCNL) have been reported. However, there are no guidelines for the most effective management of this challenging situation.

This case report shows how individual the treatment possibilities should be to become successful. We used cystolithotripsy, SWL, PCNL, URS, and open surgery to get the patient stone-free.

## Case presentation

A 26-years-old Caucasian, white woman was referred to our department of Urology to extract the former implanted ureteral stents. In reporting her history, the patient indicated that she got ureteral stents on both sides because of nephrolithiasis in June 2003. While hospital stay in April 2007 because of pregnancy the encrusted ureteral stents were found. The time of pregnancy and birth as well was without any complications. The attempt of transurethral extraction of the right ureteral stent was frustrating. The patient was reporting intermittent right flank pain.

**Figure 1:**
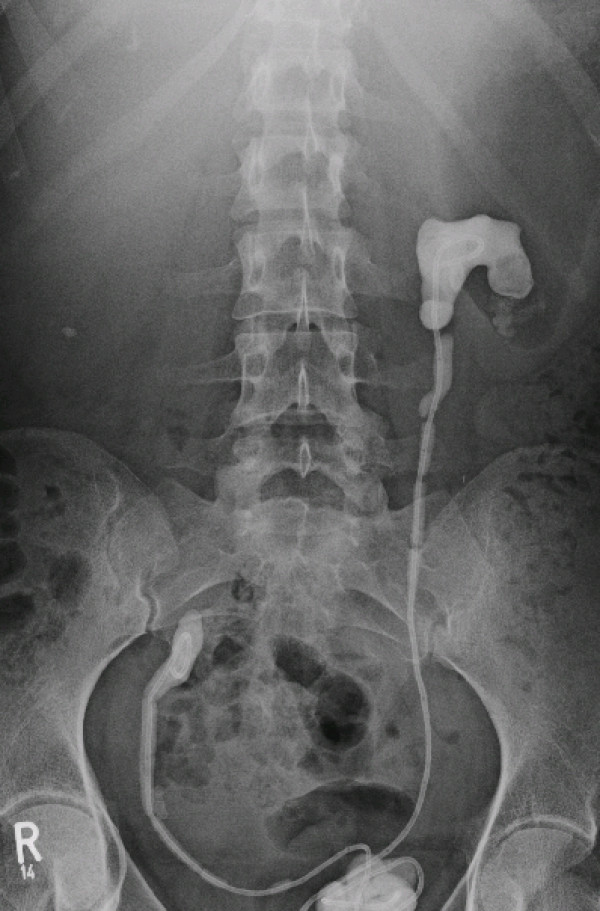
**Radiograph before therapy**.

The first presentation with the forgotten ureteral stents in our Department of Urology in June 2007 was 48 months after insertion, right one dislocated after frustrating attempt of extraction, left one with a partial staghorn calculus (figure 1). Preoperative investigations consisted of a hemogram, urine culture, serum biochemistry, abdominal ultrasonography, plain radiograph, and radionuclide renal scan. Stents were polyurethane Double-J stents. Patient anatomy, stent encrustration, and the complexity were evaluated by plain radiographs and kidney function test. The reason for stent retention was poor compliance. The kidney function test (clearance) shows a normal tubular function of 248 ml/min/1.73 m^2 ^body surface without side difference, right side hydronephrosis. Serum creatinine level was 58 μmol/l, urine culture was negative. The treatment decision was based on the clinical presentation and image finding. In this case a combination of transurethral cystolithotripsy with Lithoclast for the bladder stone, open ureterolithotomy for the dislocated right ureteral stent, and PCNL, SWL and URS for the left encrusted ureteral stent was chosen.

**Figure 2:**
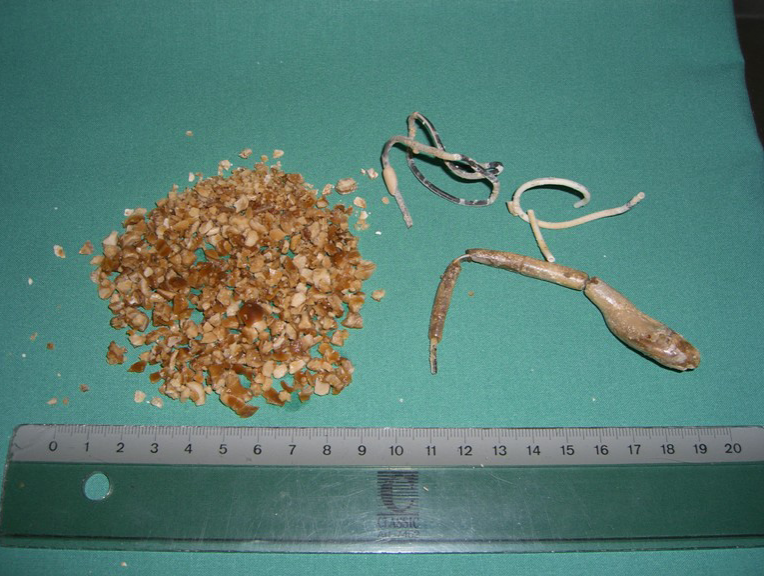
**Stone mass after Lithoclast and ureterolithotomy**.

First part of the treatment in July 2007 was transurethral disintegration of the bladder stone with the Lithoclast, abscission of the distal part of the ureteral stents at ostium level, and removal of the stones. Five days later an open ureterolithotomy for removal of the upper part of the right ureteral stent has been done. While this operation a new ureteral stent was inserted on the right side. Three months before this operation there was a frustrating attempt to remove this right stent. As it can be seen in figure 1 there is a big stone mass in the lower part of the ureter. While the transurethral operation a manipulation at the right ureteral stent was made without success as well. The whole mass of the stones and the lower part of the stents is shown in figure 2.

Second part of the treatment in September 2007 consists of PCNL and an antegrade change of the ureteral stent on the left side. PCNL was performed by only one puncture, after operation the middle and lower calyx were stone free.

Third part of the treatment in October 2007 with SWL for the left stones in the upper calyx has been done. After that URS on the left side with free ureter and extraction of both ureteral stents was performed.

**Figure 3:**
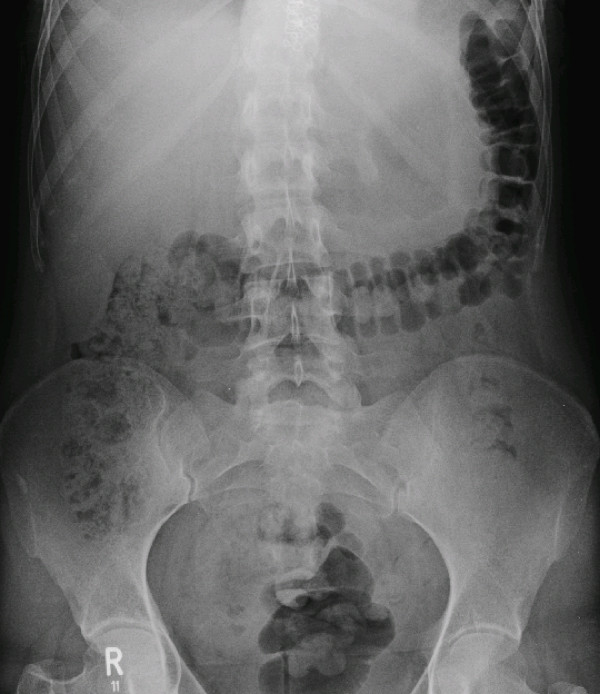
**Radiograph after therapy**.

Last part of the treatment in November 2007 was performed with PCNL on the left side, and SWL of the right side. After this treatment the patient was stone free (figure 3). The kidney function test (clearance) in January 2008 shows a normal tubular function of 264 ml/min/1.73 m^2 ^body surface without side difference, no hydronephrosis. IVU in this time shows a chronic dilated renal pelvis with a delayed flow. After a follow-up time of six months renal function remains equal and no new stone has been diagnosed.

## Discussion

Encrusted, retained stents represent a challenge for urologists and necessitate a multimodality endourologic approach. Few studies have introduced algorithms for the management of retained indwelling ureteral stents [7,8].

Clearly, there is no consensus on which method is the best for managing forgotten stents efficiently. Our approach includes a thorough preoperative imaging evaluation to decide the treatment strategy. The size of the stone burden and the site of encrustation determine the specific endourologic approach. In any case, no significant force should be used to attempt stent removal. Our plan to remove first the distal part of the stone burden with Lithoclast, and in this special case with open ureterolithotomy of the right dislocated stent, then the partial staghorn calculus with PCNL, SWL, and URS is in agreement with previous reports [6,8].

PCNL was used for the stone-covered proximal end of the stent, it seemed reasonable to go first to the distal end, manage the stone burden including the remove of the lower curl, and facilitate PCNL with placing a ureteral access catheter.

SWL is indicated only for localized, low-volume encrustrations in kidneys that have reasonably good function to allow spontaneous clearance of fragments [7]. We think that a SWL is senseful only for stones left after a therapy with PCNL as it has been mentioned in other studies before [3,4].

Although endourology can provide all necessary solutions for the management of forgotten indwelling stents, the best treatment remains prevention. It has been reported that a period between 2 and 4 months can be considered optimal [6,8]. However, patients with recurrent encrustations on stents should have them changed earlier (every 6–8 weeks). On the other hand, poor compliance of the patients represents the main cause of a forgotten stent. Education of patients and explanation of the problems that a forgotten stent can cause may convince to comply.

## Conclusion

This case shows the catastrophic, but preventable, complication to forgotten ureteral stents and the multimodal option for managing this complex case. Urologists should bear in mind the presence of severe encrustations when they have to deal with a forgotten stent. Combined urologic techniques can achieve successful and safe management of forgotten stents, but treatment should be tailored to the volume of encrustation and associated stone.

## Abbreviations

SWL: extracorporeal shock wave; PCNL: percutaneous nephrolithotomy; URS: ureterorenoscopy; IVU: intravenous urography.

## Consent

Written informed consent was obtained from the patient for publication of this case report and accompanying images. A copy of the written consent is available for review by the Editor-in-Chief of this journal.

## Competing interests

The authors declare that they have no competing interests.

## Authors' contributions

THE drafted the manuscript, performed a literature review, and was involved in the clinical follow-up. SH participated in the surgery and was involved in the clinical follow-up. JR participated in the surgery, was involved in the clinical follow-up, and supervised this report. All authors read and performed the clinical follow-up.
